# Comparison of Free-Space and Waveguide-Based SERS Platforms

**DOI:** 10.3390/nano9101401

**Published:** 2019-10-01

**Authors:** Nina Turk, Ali Raza, Pieter Wuytens, Hans Demol, Michiel Van Daele, Christophe Detavernier, Andre Skirtach, Kris Gevaert, Roel Baets

**Affiliations:** 1 Photonics Research Group, Department of Information Technology, Ghent University-IMEC, Technologiepark 126, 9052 Ghent, Belgium; 2 Center for Nano- and Biophotonics, 9052 Ghent, Belgium; 3 IMEC, 3001 Leuven, Belgium; 4 VIB-UGent Center for Medical Biotechnology, 9000 Ghent, Belgium; 5 Department of Biomolecular Medicine, Ghent University, 9000 Ghent, Belgium; 6 CoCooN Research Group, Department of Solid State Sciences, Ghent University, 9000 Ghent, Belgium; 7 NanoBioTechnology Laboratory, Department of Biotechnology, Ghent University, 9000 Ghent, Belgium

**Keywords:** Raman spectroscopy, SERS, photonic integrated circuit, waveguide-based SERS, nanoplasmonic slot waveguide, gold nanodomes, peptide detection

## Abstract

Surface-Enhanced Raman Spectroscopy (SERS) allows for the highly specific detection of molecules by enhancing the inherently weak Raman signals near the surface of plasmonic nanostructures. A variety of plasmonic nanostructures have been developed for SERS signal excitation and collection in a conventional free-space microscope, among which the gold nanodomes offer one of the highest SERS enhancements. Nanophotonic waveguides have recently emerged as an alternative to the conventional Raman microscope as they can be used to efficiently excite and collect Raman signals. Integration of plasmonic structures on nanophotonic waveguides enables reproducible waveguide-based excitation and collection of SERS spectra, such as in nanoplasmonic slot waveguides. In this paper, we compare the SERS performance of gold nanodomes, in which the signal is excited and collected in free space, and waveguide-based nanoplasmonic slot waveguide. We evaluate the SERS signal enhancement and the SERS background of the different SERS platforms using a monolayer of nitrothiophenol. We show that the nanoplasmonic slot waveguide approaches the gold nanodomes in terms of the signal-to-background ratio. We additionally demonstrate the first-time detection of a peptide monolayer on a waveguide-based SERS platform, paving the way towards the SERS monitoring of biologically relevant molecules on an integrated lab-on-a-chip platform.

## 1. Introduction

Raman spectroscopy enables for the highly specific detection of molecules by probing their vibrational states. Such Raman signals are, however, inherently weak. In the last decades, several methods have been developed to enhance Raman signals, such as coherent anti-Stokes scattering that relies on non-linear Raman scattering [1] or Surface-Enhanced Raman Spectroscopy (SERS) [2,3,4,5,6,7,8,9,10,11,12,13,14,15]. Two types of SERS enhancement mechanisms have been proposed to explain the SERS effect—the electromagnetic (EM) mechanism and the chemical enhancement (CE) mechanism [16,17,18,19]. The EM enhancement, where the local EM field is greatly increased near plasmonic nanostructures by localized surface-plasmon resonances, is thought to be the main contribution to the SERS enhancement [19]. On the other hand, the CE enhancement is characterized by the shifting of the Raman scattering in non-resonance to that in resonance through the formation of charge transfer complexes between adsorbed molecules and metal surfaces, and its contribution is thought to be far smaller than the EM enhancement [19]. In this paper, we will focus solely on the EM mechanism of SERS enhancement. A variety of plasmonic nanostructures providing high SERS enhancements have been developed for SERS signal excitation and collection in a conventional free-space confocal microscope [20]. Colloidal metal nanoparticles enable strong SERS enhancements, yet their fabrication methods offer only limited control of their geometry, size and position, consequently affecting the reproducibility of SERS measurements [20]. Conversely, a variety of SERS substrates has been developed using top-town fabrication techniques such as nanosphere lithography [21,22,23,24], and deep-UV [11] and electron beam lithography [25,26]. These techniques enable precise control of the shape and position of the nanostructures, which allows more tunable and reproducible SERS enhancements [20]. Among the top-down fabricated SERS substrates, the gold nanodomes offer one of the highest SERS enhancements [14,15]. Their fabrication is simple and scalable, while also ensuring better control of the hotspot size and enhancement factor as compared to colloidal approaches. 

Moreover, novel techniques for the more efficient collection of Raman signals have recently emerged as alternatives to the conventional Raman microscope, such as hollow-core photonic crystal fibers [27] and nanophotonic waveguides on photonic integrated circuits (PICs) [28,29,30,31]. Nanophotonic waveguides can be used to efficiently excite and collect the Raman signal of the molecules close to the waveguide, opening up the possibility of bringing the selectivity of Raman measurements to an integrated lab-on-a-chip platform. PICs have been also used to collect SERS signals from external colloidal metal nanoparticles [32,33,34]. However, to enable reproducible waveguide-based excitation and collection of SERS spectra, top-down fabricated plasmonic structures can be integrated on dielectric nanophotonic waveguides. In the first demonstration of waveguide-excited and collected SERS on integrated plasmonic nanostructures, gold bowtie antennas were patterned on a silicon nitride waveguide in a two-step electron beam lithography process [35,36]. Electron beam lithography is, however, resource-intensive and time-consuming, so an alternative nanosphere lithography approach was used to fabricate integrated nanotriangles. [37] Besides easier fabrication, the integrated nanotriangles also achieved a significant improvement in SERS enhancement compared to the integrated bowties. More recently, new waveguide-based SERS platforms have emerged, such as the nanoplasmonic slot waveguide [38,39,40,41] and the nanoporous gold on suspended silicon nitride waveguides [42]. 

The on-chip SERS platform shows great potential for high-throughput SERS assays on low sampling volumes, which is especially relevant for the detection of biological molecules. However, most biological molecules have low Raman cross-sections, making the SERS enhancements of the integrated bowties and integrated nanotriangle platforms insufficient for their detection. The high SERS enhancement of the nanoplasmonic slot waveguide might, however, prove sufficient for the SERS detection of biological molecules [38]. In addition to high SERS enhancement, the nanoplasmonic slot waveguides are fabricated using a combination of atomic layer deposition and deep UV photolithography, enabling mass scale manufacturing. Furthermore, they offer a non-resonant enhancement, making the SERS enhancement independent of excited and scattered wavelengths. 

In this paper, we evaluate the SERS performance of the two top-performing SERS substrates, one for free-space and other for the waveguide-based excitation and collection of SERS signals. This comparison is highly relevant for future on-chip SERS applications. We compare the gold nanodomes in which the signal is excited and collected in free space, and the waveguide-based nanoplasmonic slot waveguides. We examine the SERS signal enhancement and the SERS background of the different SERS platforms using a monolayer of p-nitrothiophenol (NTP). We additionally demonstrate the first-time detection of a biomolecule on a waveguide-based SERS platform, paving the way towards SERS detection and the monitoring of biologically relevant molecules on an integrated lab-on-a-chip platform.

## 2. Materials and Methods

### 2.1. Fabrication of SERS Substrates

**Figure 1:**
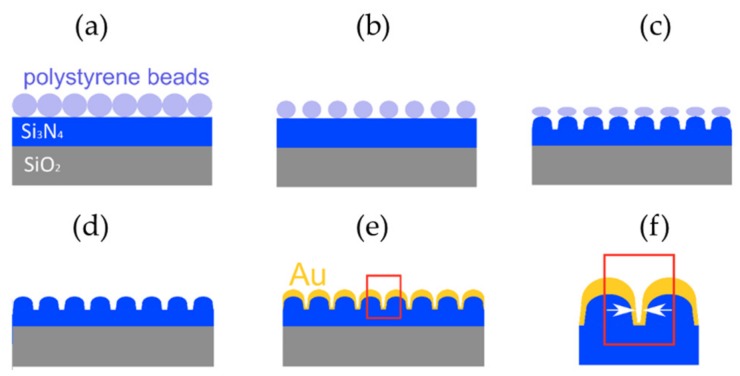
Fabrication process of gold nanodomes. (**a**) Spincoating of polystyrene beads on a Si_3_N_4_ chip. (**b**) Thinning down of the polystyrene beads by oxygen plasma. (**c**) Etching of the nanodome pattern in Si_3_N_4_. (**d**) Removal of the remains of the polystyrene beads. (**e**) Gold deposition. (**f**) The arrows mark the gap in the gold nanodomes.

Gold nanodomes were fabricated using a nanosphere lithography (NSL) based process, described in detail in [15] and shown in Figure 1. Briefly, we started from a 200 nm thick film of silicon nitride (Si_3_N_4_) deposited on top of a 4-inch silicon wafer using PECVD deposition (Vision 310-PECVD, AdvancedVacuum, Uppsala, Sweden). We spin-coated a colloidal solution of 450 nm polystyrene beads (microParticles GmbH, Berlin, Germany) on the Si_3_N_4_ surface, thereby generating a monolayer of hexagonally-close packed polystyrene beads. The polystyrene beads were then thinned down in an oxygen plasma (GIGA batch 310 M, PVA-TePla, Wettenberg, Germany), and a periodic pattern of nanodomes was etched into the Si_3_N_4_ substrate by an anisotropic reactive-ion etch (Vision 320-RIE, AdvancedVacuum, Uppsala, Sweden). These two steps effectively determined the height and the width of the gap between the nanodomes, the two most important parameters for tuning the plasmonic resonance and thus, the SERS enhancement factor [15]. The remains of the polystyrene beads were lifted off in dichloromethane (Sigma-Aldrich, Overijse, Belgium) and the wafers were cleaned in a piranha solution (H_2_SO_4_:H_2_O_2_, 3:1; purchased from Sigma-Aldrich, Overijse, Belgium). Finally, a 2 nm thick titanium adhesion layer and a 130 nm thick gold layer were sputtered onto the sample (Alcatel SCM600, Bittmann applied technologies, Rotenburg, Germany). The nanodomes were characterized with scanning electron microscopy (SEM; Nova 600 Nanolab, FEI, Hillsboro, OR, USA) and the nanodome gap size was determined to be 12 ± 2 nm. UV-VIS reflection and SERS measurements were performed to optimize their localized surface-plasmon resonance wavelength in order to match the 785 nm laser used for the SERS excitation, as published in [14]. 3D Finite-Difference Time-Domain (FDTD) simulations predict the maximum SERS enhancement factor of the gold nanodomes for a single molecule in the order of 10^7^ [15]. 

**Figure 2:**
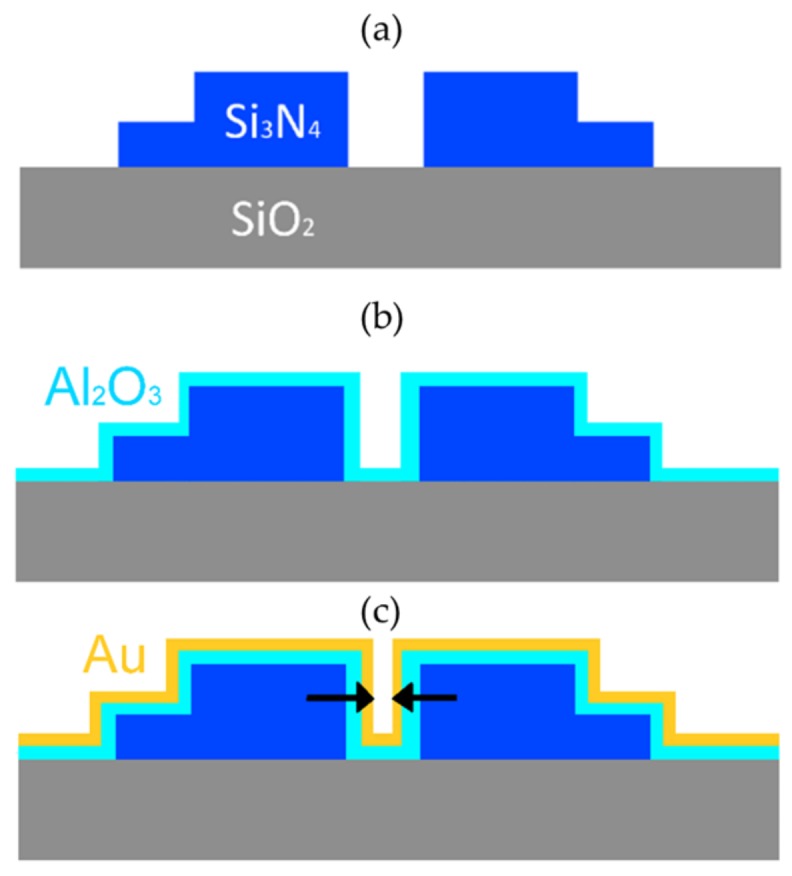
Fabrication of a nanoplasmonic slot waveguide. (**a**) Fabrication of the Si_3_N_4_ slot waveguides. (**b**) Atomic layer deposition of Al_2_O_3_. (**c**) Gold deposition. The arrows mark the gap width of the nanoplasmonic slot waveguide.

Nanoplasmonic slot waveguides were fabricated using a combination of atomic layer deposition and deep UV photolithography, as described in detail in [38] and shown in Figure 2. Firstly, 2.3 µm thick SiO_2_ and 220 nm thick Si_3_N_4_ layers were deposited on a 200 nm silicon wafer. The Si_3_N_4_ slot waveguides were patterned with 193 nm deep UV optical lithography and subsequently etched by a reactive-ion etch process (fabricated by IMEC, Leuven, Belgium). The resulting average slot width was 150 nm. The minimal width of the slot is limited to 150 nm by the resolution of the deep-UV lithography. To narrow down the slot width, the waveguides were uniformly coated with 58 nm Al_2_O_3_ by using atomic layer deposition (ALD; deposited on the home-built ALD setup, Ghent University, Ghent, Belgium) that has a low SERS background [43]. After ALD, gold waveguides were defined using photolithography (MA6 mask aligner, SUSS MicroTec, Garching, Germany), and a 2 nm titanium adhesion layer and a 15 nm thick layer of sputtered gold were deposited. The nanoplasmonic slot waveguides were characterized with scanning electron microscopy and the gap size was determined to be 15 ± 0.5 nm. Due to the technical limitations of the setup, UV-VIS reflection spectra of the waveguide-based SERS substrates could not be measured. To prove the non-resonant enhancement of the nanoplasmonic slot waveguides, we relied on the simulation results [38,44], which are supported by the experimental measurements of the SERS signal at two different laser excitation wavelengths (632 and 785 nm), as reported in [38]. Numerical simulations predicted the maximum SERS enhancement factor of the nanoplasmonic slot waveguide for a single molecule to be 1.5 × 10^7^ [38]. 

### 2.2. Acquisition of SERS Spectra of P-Nitrothiophenol 

Both SERS substrates were functionalized with a self-assembled monolayer of p-nitrothiophenol (NTP; purchased from Sigma-Aldrich, Overijse, Belgium) that selectively binds to the gold via a gold-thiol bond, as described in detail in [38]. In short, the SERS substrates were cleaned with acetone, isopropyl alcohol and deionized (DI) water, and then were dried with a N_2_ gun. After the oxygen plasma treatment, they were immersed overnight in 1 mM NTP solution in ethanol, and then rinsed with ethanol to remove non-bound NTP.

**Figure 3:**
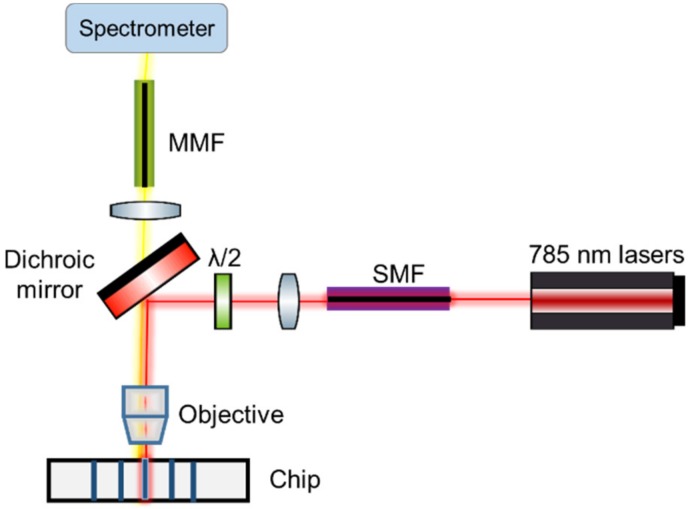
Schematic of an optical setup used to measure the surface-enhanced Raman spectra. Gold nanodomes were measured in a conventional free-space configuration, whereas in the case of the nanoplasmonic slot waveguides, the microscope objective was used to couple the light to the waveguide and then to collect the SERS signal in the back reflection.

SERS spectra of the two substrates were acquired on a confocal Raman microscope (Alpha 300 R+, WITec, Ulm, Germany) equipped with a −65 °C to −70 °C cooled CCD camera (iDus 401BR-DD, Andor, Belfast, UK) and a 785 nm diode laser (XTRA II, Toptica, Graefelfing, Germany). Stokes scattered light was collected by a 100 μm diameter multimode fiber and fed to the spectrometer, which used a 600 l/mm grating to diffract the Stokes scattered light on the spectral camera. All lines of the CCD camera were read out (using full vertical binning), using a vertical shift speed of 16.25 μs and a horizontal shift speed of 0.033 MHz. For the gold nanodomes, a 300 µW laser power before the microscope objective and a Nikon PlanFluor 10×/0.3 objective (Nikon, Tokyo, Japan) were used to acquire spectra on a spatially distributed map of 10 × 10 pixels in a 20 × 20 µm area with an integration time of 0.13 s on each point to avoid signal degradation upon laser illumination. Each trace represents a median spectrum of one map. To acquire the SERS spectra of the nanoplasmonic slot waveguide on the confocal microscope, the sample was positioned vertically and end-fire coupled, as shown in Figure 3 and explained in more detail in [38]. The polarization of the 785 nm excitation beam was set to the TE mode of the waveguide and a Zeiss 100×/0.9 EC Epiplan NEOFLUAR:∞/0 objective (Carl Zeiss AG, Oberkochen, Germany) was used to couple the light into the waveguide with the laser power of 350 µW measured before the microscope objective. The integration time was set to 1 s. The SERS signal was collected in back reflection using the same objective. The objective and chip were aligned with 100 nm accuracy based on a maximum intensity of the waveguide Raman spectrum. Simultaneously, maximum light scattering along the waveguide was observed from a camera imaging the top surface of the chip.

### 2.3. Acquisition of SERS Spectra of a Peptide

Both SERS substrates were functionalized with a self-assembled monolayer of the in-house made peptide NH_2_-CALNNF_CN_SF_CN_GGGGVRGNFSF-COOH, where each letter represents a natural amino acid and F_CN_ represents a non-natural amino acid cyano-phenylalanine. A similar peptide has been previously used to demonstrate the detection of the protease activity on a gold nanodome platform such as in [15], where the fabrication and the labeling are described in detail. Briefly, the in-house synthesized peptide was first dissolved in dimethylformamide (Sigma-Aldrich, Overijse, Belgium) and then diluted to the concentration of 100 µM/ml in 10% acetonitrile/water solution. The SERS substrates were cleaned with acetone, isopropyl alcohol and DI water, and then were dried with N_2_ gun. After the oxygen plasma treatment, the samples were immersed overnight in the peptide solution, and then rinsed with deionized water to remove any peptides that did not covalently bind to the gold. The sample was placed in a Petri dish filled with 3 ml of 50 mM ammonium bicarbonate buffer (pH 7.8; purchased from Sigma-Aldrich, Overijse, Belgium). The SERS spectra of the peptide were acquired with the same confocal microscope that was used for the detection of NTP spectra, as described in the previous paragraph. For gold nanodomes, a 1 mW laser power before the microscope objective and a 40×/0.5 Zeiss water immersion objective (Carl Zeiss AG, Oberkochen, Germany) were used to acquire spectra on a spatially distributed map of 7 × 7 pixels in a 40 × 40 µm area with an integration time of 3 s on each point to avoid signal degradation upon laser illumination. Each trace represents a median spectrum of one map. For the nanoplasmonic slot waveguides, we used 1 mW laser power before the microscope objective and a Zeiss ×/1.0 W-Plan Apochromat ∞/0 objective (Carl Zeiss AG, Oberkochen, Germany) to acquire 10 consecutive spectra with an integration time of 10 s each.

## 3. Results and Discussion

### 3.1. Comparison of Free-Space Excited Gold Nanodomes and Waveguide-Based Nanoplasmonic Slot Waveguide

In this section, we examine the fabrication processes and the SERS performance of gold nanodomes, where the signal is excited and collected in free space, and waveguide-based nanoplasmonic slot waveguide. 

#### 3.1.1. Scalability of the Fabrication Processes

**Figure 4:**
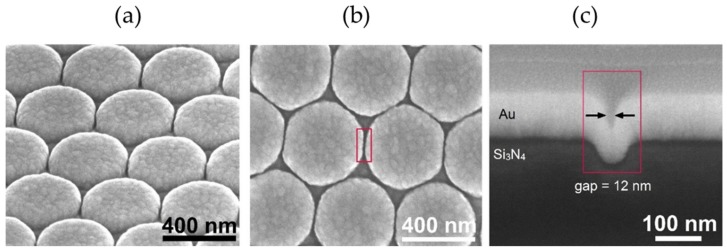
Scanning electron microscope images of gold nanodomes. (**a**) Tilted view. (**b**) Top-down view. (**c**) Cross-section of a nanodome-patterned chip with a 12 nm wide gap between nanodomes.

The SEM images of the gold nanodomes are shown in Figure 4. The fabrication of gold nanodomes is simple and scalable. Many SERS substrates are fabricated using electron beam lithography; however, this resource-intensive and time-consuming method is primarily used for prototyping and is not often used in industrial scale fabrication. On the other hand, the fabrication process based on nanosphere lithography is easily implemented in a mass scale production, making gold nanodomes interesting for industrial applications. 

**Figure 5:**
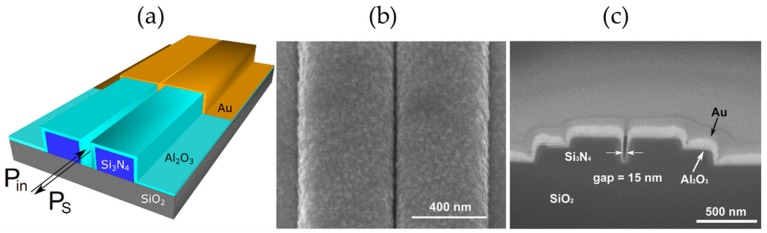
Nanoplasmonic slot waveguide. (**a**) Schematic showing that the input and Stokes powers are guided by the waveguide. (**b**) Scanning electron microscope image of the gold-covered slot in top view. (**c**) Cross-section of a nanoplasmonic slot waveguide with a gap of 15 nm.

Figure 5 shows the schematic and the SEM images of the nanoplasmonic slot waveguide. The nanoplasmonic slot waveguide provides high SERS enhancement that outperforms previously developed waveguide-based SERS platforms, such as integrated bowties [35,36] and integrated nanotriangles [37]. In addition to high SERS enhancement, the nanoplasmonic slot waveguides are fabricated using a combination of atomic layer deposition and deep UV photolithography, enabling mass scale manufacturing.

Both gold nanodomes and nanoplasmonic slot waveguides are, therefore, fabricated using widely available, mass-scalable fabrication methods that make them interesting for industrial applications.

#### 3.1.2. SERS Performance Comparison

To compare the SERS performance of gold nanodomes and nanoplasmonic slot waveguides, we used a monolayer of p-nitrothiophenol (NTP). NTP shows prominent SERS peaks at the Raman shifts of 1110, 1339 and 1573 cm^−1^ that correspond to C–S stretching, symmetric nitro stretching and phenyl ring modes of the nitrothiophenol molecule [45]. We used the integrated counts of the 1339 cm^−1^ NTP mode as a metric of the SERS signal and the SERS background. We additionally assessed the coefficient of variation on the SERS signal across a single chip on the gold nanodomes to be 6–7%, as previously reported in [15]. For the metal slot waveguides, this coefficient cannot be defined since we are exciting the SERS response through a single access waveguide. 

**Figure 6:**
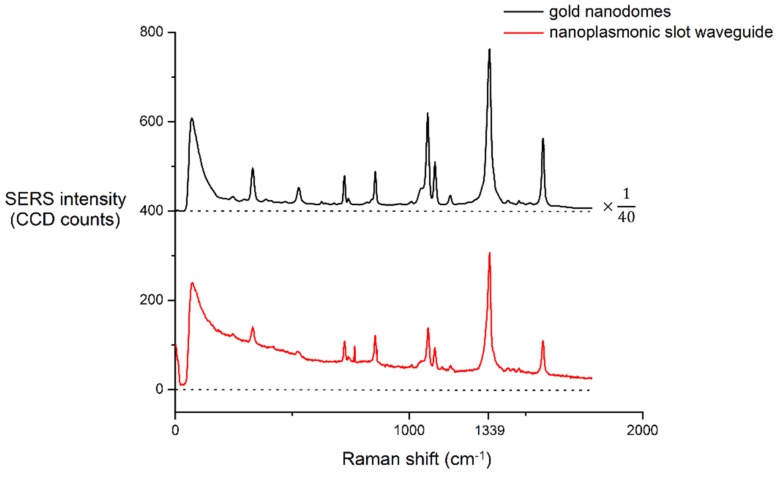
Averaged SERS spectra of the NTP monolayer acquired on gold nanodomes and on the nanoplasmonic slot waveguide. The spectrum on the gold nanodomes was obtained using a laser power of 300 µW and an integration time of 0.13 s. The spectrum on the nanoplasmonic slot waveguide was obtained using a laser power of 350 µW and an integration time of 10 s. The SERS spectrum on the nanodomes was divided by a factor of 40 to allow for better visualization. We subtracted the dark counts, but not the SERS background of the spectra. The spectra are offset on the y-axis for clarity, and the dashed line represents the zero line of each spectrum.

We acquired the SERS spectra of the gold nanodomes and nanoplasmonic slot waveguides using different excitation laser powers and integration times. These parameters were optimized so that no photoinduced reduction of NTP to dimercaptoazobenzene was observed, since these chemical changes would affect the SERS signal strength of the 1339 cm^−1^ mode of the NTP [24]. On Figure 6, we show the averaged SERS spectra of the NTP monolayer acquired on gold nanodomes and on the nanoplasmonic slot waveguide. The first step to consistent comparison of the SERS signals was to normalize the SERS signal strength on the integration time. Since the signal increases linearly with the integration time, we simply normalized the SERS signal strength to the integration time of 1 s. To include the effect of the different excitation laser powers, we calculated the ratio of total collected Stokes power *P_S_* over input power *P_in_* as:

(1) $$\eta =\;\frac{P_{S}}{P_{in}},$$

where *P_in_* is the laser power that is used to excite the SERS response of our analyte. In the case of nanodomes, all the free-space laser power is used to excite the SERS response of our analyte, so *P_in_* is the laser power coming out of the microscope objective, whereas in the waveguide-based SERS platforms, we define *P_in_* as the laser power guided in the waveguide to the plasmonic structure, as shown in Figure 5a. Stokes power *P_S_* is the power of the SERS scattered photons, which in our case was at the 1339 cm^−1^ peak. To convert the measured CCD counts to *P_S_*, we use the formula:

(2) $$P_{S}=F_{ph}h\left(\nu_{0}-\;\nu_{S}\right)T_{m}^{-1}.$$

**Figure 7:**
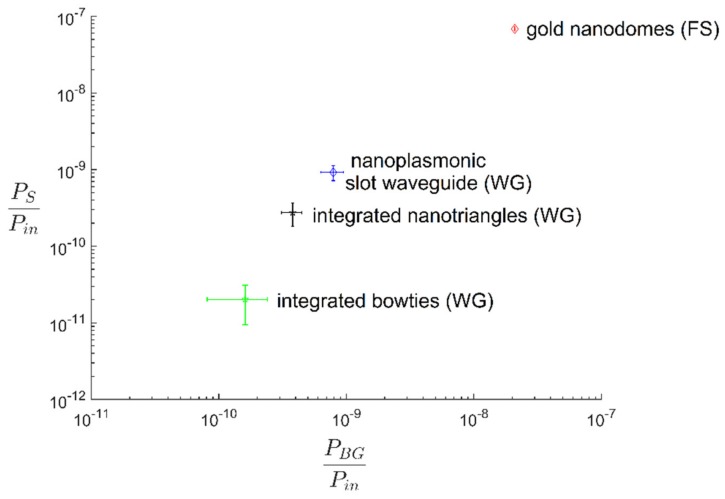
Comparison of SERS background power *P_BG_* (x-axis) and SERS Stokes power *P_S_* (y-axis) of different SERS platforms. Both parameters are normalized on the input power and the integration time. FS indicates free-space and WG is the waveguide-based excitation and collection of the SERS signal.

For the 1339 cm^−1^ NTP peak excited at 785 nm, the Stokes frequency (*ν_0_ − ν_S_*) equals 342 THz and the transmission of the microscope *T_m_* is 0.61 at the 870 nm Stokes shifted wavelength. For the specific case of our spectrometer with settings as described in Methods and Materials, the photon flux *F_ph_* equals 5.9 times the number of counts in the Raman spectrum. The same calculation was also applied to calculate the SERS background signal. In Figure 7, the performance of the gold nanodomes and nanoplasmonic slot waveguides is compared in terms of the SERS signal strength and the SERS background. Additionally, we included the SERS performances of integrated bowties [35] and integrated nanotriangles [37] in the graph to highlight the progress that has been achieved in the last few years in the field of waveguide-based SERS platforms. We see that the free-space excited gold nanodomes offer very high SERS enhancements; however, their high background contribution limits their SERS performance. On the other hand, the nanoplasmonic slot waveguides provide lower SERS enhancements, yet their SERS background is reduced compared to that of the nanodomes. 

SERS background is still not sufficiently understood, and in the last few years, several new models were proposed to describe its origins [46,47,48,49]. Exact identification of the origin of the SERS background in gold nanodomes and nanoplasmonic slot waveguides is, therefore, a difficult task that is beyond the scope of this paper. We can, however, try to narrow down the possible origins of the SERS background. The SERS background was experimentally linked to the strength of the localized surface plasmons, the identity of the adsorbate and adsorbate coverage [46]. In our case, we use the same adsorbate (NTP) under identical labeling conditions to characterize both the nanodomes and the nanoplasmonic slot waveguides. Furthermore, we always use newly fabricated SERS substrates in order to avoid any contaminants from previous labelings. We thus argue that the differences in the SERS background probably do not arise from the adsorbate itself or the contamination of our samples. Conversely, the effects of plasmons on the SERS background are harder to evaluate. We fabricated the structures using the same gold deposition technique, indicating that the differences in the SERS background did not originate in the difference of the gold deposition. SERS enhancement was then achieved via localized surface-plasmon resonance in gold nanodomes [15] and via propagating the surface plasmon polariton in the nanoplasmonic slot waveguide [38], which might at least in part cause the differences in the SERS background. Additional differences in the SERS background might also have arisen from the two different ways of exciting the SERS response, that is via free-space and waveguide-based excitation for the gold nanodomes and nanoplasmonic slot waveguides, respectively. In the case of the nanoplasmonic slot waveguide, the silicon nitride waveguides might have provided some additional contribution to the SERS background.

To compare the different SERS platforms, we considered both the SERS enhancement and SERS background in one figure of merit. We propose signal-to-background ratio (SBR) and signal-to-noise ratio (SNR) as relevant figures for comparison of the SERS performance of different platforms. In the case of strong SERS signals compared to the background, the accuracy of the SERS signal analysis was limited by the imperfect background subtraction, since the background needed to be estimated from algorithmic extrapolation or from separate measurements [50]. In this case, we could use SBR as metric to evaluate the SERS performance:

(3) $$SBR=\;\frac{P_{S}}{P_{BG}}=\;\frac{\frac{P_{S}}{P_{in}}}{\frac{P_{BG}}{P_{in}}}.$$

The SBR of different SERS platforms are shown in Table 1. We see that the nanoplasmonic slot waveguide offers a significant improvement compared to other waveguide-based SERS platforms. It provided more than 10-times higher SBR than integrated bowties and 60% higher SBR than integrated nanotriangles, respectively. The nanoplasmonic slot waveguide, therefore, approached the SBR of the free-space excited gold nanodomes, providing only 3-times lower SBR than the gold nanodomes. 

On the other hand, when the photon number of the total signal within the integration time was below the critical level, the accuracy of the SERS signal analysis was not limited by the imperfect accuracy of the background subtraction, but rather by shot noise. SNR then provided a more relevant metric for the SERS performance of different SERS platforms [50]. The noise N, due to shot noise, was proportional to the square root of the sum of the SERS peak and the SERS background signal. In the case of low SERS signals, the main contribution to the noise N (being the rms value of the signal fluctuation) is, therefore, from the background signal:

(4) $$N=\sqrt{\frac{P_{BG}t}{h\nu }}.$$

The SNR is then defined as:

(5) $$SNR=\frac{\frac{P_{S}t}{h\nu }}{\sqrt{\frac{P_{BG}t}{h\nu }}}=\frac{\frac{P_{S}}{P_{in}}}{\sqrt{\frac{P_{BG}}{P_{in}}}}\sqrt{\frac{P_{in}t}{h\nu }}.$$

We list the SNR of the different SERS platforms in Table 1. Since the SNR also depends on the square root of the input power *P_in_* and integration time *t*, we set the input power at 1 mW and the integration time to 1 s to be able to do a relative comparison of the SNR of the different SERS platforms. If we use another input power, the absolute value of SNR will change, but the relative SNR of different SERS platforms will remain the same. We see that the nanoplasmonic slot waveguides provide significant SNR improvements compared to the other waveguide-based SERS platforms. Their SNR ratio is 20-times better than that for integrated bowties and 2-times better than in the case of integrated nanotriangles. Nevertheless, free-space excited gold nanodomes still outperform the nanoplasmonic slot waveguide with 15-times higher SNR. 

Besides the improved SBR, the nanoplasmonic slot waveguide also offers non-resonant SERS enhancement, making the SERS enhancement independent of excited and scattered wavelengths, which offers an advantage when compared to the highly resonant gold nanodomes. Moreover, the waveguide-based SERS platform shows great potential for high-throughput SERS assays on low sampling volumes, and is especially relevant for the detection of biological molecules.

### 3.2. SERS Detection of a Peptide on a Waveguide-Based Platform

A waveguide-based SERS platform offers several advantages over conventional free-space SERS substrates for biological applications. The waveguide-based SERS platform allows parallel measurements of a large number of SERS analytes, enabling high-throughput assays that are of particular interest for pharmacological drug discovery. Furthermore, the efficient SERS enhancement mechanisms allow for the measurement to be performed on low sampling volumes, additionally minimizing the cost of such assays. 

To demonstrate the detection of a biomolecule on a waveguide-based SERS platform for the first time, we chose the peptide NH_2_-CALNNF_CN_SF_CN_GGGGVRGNFSF-COOH. Here each letter represents a natural amino acid and F_CN_ represents a non-natural amino acid cyano-phenylalanine. A similar peptide containing only natural amino acids has been previously used to demonstrate the detection of protease activity on the gold nanodome platform [15]. Proteases are enzymes that catalyze the hydrolysis of peptide bonds and that therefore play important roles in various human diseases [51]. A real-time, multiplexed method for detection of protease activity is, therefore, important for the development of new drugs that, for instance, could inhibit disease-associated proteases.

In the peptide NH_2_-CALNNF_CN_SF_CN_GGGGVRGNFSF-COOH, the SERS signal originates from the aromatic amino acids phenylalanine and cyano-phenylalanine. We observe the SERS peak of phenylalanine at 1003 cm^−1^ assigned to the trigonal ring breathing of the benzene ring [52], and the peak of cyano-phenylalanine at 1180 cm^−1^. To additionally increase the Raman cross-section of the peptide, we incorporated doubled aromatic amino acids in the peptide, effectively doubling the Raman cross-section independently of the SERS platform that is used to detect the peptide.

**Figure 8:**
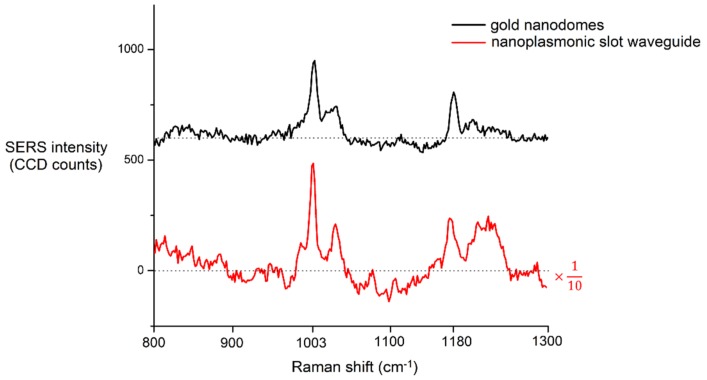
Background-subtracted SERS spectra of the peptide NH_2_-CALNNF_CN_SF_CN_GGGGVR-GNFSF-COOH acquired on gold nanodomes and on the nanoplasmonic slot waveguide. The spectrum on the gold nanodomes was obtained using a laser power of 1 mW and an integration time of 1 s. The spectrum on the nanoplasmonic slot waveguide was obtained using a laser power of 1 mW and an integration time of 30 s, and the SERS intensity was divided by a factor of 10 to allow for better visualization. The spectra are offset on the y-axis for clarity, and the dashed line represents the zero line of each spectrum.

We first detected the SERS spectra of the peptide on the gold nanodomes. Next, we obtained the SERS spectra of the peptide on the nanoplasmonic slot waveguide, which is, to the best of our knowledge, the first-time detection of a biomolecule on a waveguide-based SERS platform. Both spectra are shown in Figure 8. 

Previously developed waveguide-based SERS platforms, such as integrated bowties [35] and integrated nanotriangles [37], have been able to detect organic molecules with relatively high Raman cross-sections, such as NTP. However, their SERS enhancements were not high enough to detect biological molecules with low Raman cross-sections. We have used nanoplasmonic slot waveguide in combination with the peptide with doubled aromatic amino acids that intrinsically increase its Raman cross-section in order to detect a biological molecule for the first time on a waveguide-based SERS platform, paving the way towards SERS detection and the monitoring of biologically relevant molecules on an integrated lab-on-a-chip platform.

## 4. Conclusions

In this paper, we evaluated the SERS performance of gold nanodomes in which the signal is excited and collected in free space and in waveguide-based nanoplasmonic slot waveguides. Both SERS platforms are fabricated using simple and scalable fabrication methods, making them interesting candidates for industrial applications. We compared the SERS signal enhancement and the SERS background of the different platforms using a monolayer of p-nitrothiophenol. We showed that the SERS enhancement of the gold nanodomes is higher than in the nanoplasmonic slot waveguide; however, their SERS performance is limited by the high SERS background. We demonstrated that the nanoplasmonic slot waveguide approaches the performance of the free-space excited gold nanodomes in terms of the signal-to-background ratio, showing great promise for future waveguide-based SERS applications. 

Combining the improved SERS signal-to-background ratio of the nanoplasmonic slot waveguide with the possibility of parallel measurements of a large number of SERS analytes on a lab-on-a-chip platform is especially interesting for biological applications. We, therefore, additionally demonstrated the first-time detection of a peptide monolayer on a waveguide-based SERS platform, paving the way towards SERS detection and the monitoring of monolayers of biologically relevant molecules on an integrated lab-on-a-chip platform. 

## Figures and Tables

**Table 1:** The signal-to-background ratio (SBR) and signal-to-noise ratio (SNR) values at *P_in_* = 1 mW and *t* = 1 s for different SERS platforms evaluated based on the 1339 cm^−1^ mode of a NTP monolayer. If we use another input power or integration time, the absolute value of the SNR will change, but the relative SNR of different SERS platforms will remain the same. FS indicates free-space and WG is the waveguide-based excitation and collection of the SERS signal. For the sake of this table, only the shot noise contribution from the background is taken into account (as it will be more relevant when looking for weaker peaks than the 1339 cm^−1^ mode).

SERS Platform	SBR	SNR (P_in_ = 1 mW, t = 1 s)
Gold nanodomes (FS)	3.28	3 × 10^4^
Integrated bowties (WG)	0.12	10^2^
Integrated nanotriangles (WG)	0.72	10^3^
Nanoplasmonic slot waveguide (WG)	1.16	2 × 10^3^

## References

[1] Yampolsky S., Fishman D.A., Dey S., Hulkko E., Banik M., Potma E.O., Apkarian V.A. (2014). Seeing a single molecule vibrate through time-resolved coherent anti-Stokes Raman scattering. Nat. Photonics.

[2] Anker J.N., Hall W.P., Lyandres O., Shah N.C., Zhao J., Van Duyne R.P. (2008). Biosensing with plasmonic nanosensors. Nat. Mater..

[3] Willets K.A., Van Duyne R.P. (2007). Localized surface plasmon resonance spectroscopy and sensing. Annu. Rev. Phys. Chem..

[4] Halas N.J., Lal S., Chang W.S., Link S., Nordlander P. (2011). Plasmons in strongly coupled metallic nanostructures. Chem. Rev..

[5] Giannini V., Fernández-Domínguez A.I., Heck S.C., Maier S.A. (2011). Plasmonic nanoantennas: Fundamentals and their use in controlling the radiative properties of nanoemitters. Chem. Rev..

[6] Chu Y., Banaee M.G., Crozier K.B. (2010). Double-resonance plasmon substrates for surface-enhanced Raman scattering with enhancement at excitation and Stokes frequencies. ACS Nano.

[7] Ye J., Wen F., Sobhani H., Lassiter J.B., Van Dorpe P., Nordlander P., Halas N.J. (2012). Plasmonic nanoclusters: Near field properties of the fano resonance interrogated with SERS. Nano Lett..

[8] Bontempi N., Carletti L., De Angelis C., Alessandri I. (2016). Plasmon-free SERS detection of environmental CO_2_ on TiO_2_ surfaces. Nanoscale.

[9] Gallinet B., Siegfried T., Sigg H., Nordlander P., Martin O.J.F. (2013). Plasmonic radiance: Probing structure at the Angström scale with visible light. Nano Lett..

[10] Siegfried T., Ekinci Y., Martin O.J.F., Sigg H. (2013). Gap plasmons and near-field enhancement in closely packed sub-10 nm gap resonators. Nano Lett..

[11] Li J., Chen C., Jans H., Xu X., Verellen N., Vos I., Okumura Y., Moshchalkov V.V., Lagae L., Van Dorpe P. (2014). 300 mm Wafer-level, ultra-dense arrays of Au-capped nanopillars with sub-10 nm gaps as reliable SERS substrates. Nanoscale.

[12] Seok T.J., Jamshidi A., Eggleston M., Wu M.C. (2013). Mass-producible and efficient optical antennas with CMOS-fabricated nanometer-scale gap. Opt. Express.

[13] Schlücker S. (2014). Surface-enhanced Raman spectroscopy: Concepts and chemical applications. Angew. Chem. Int. Ed. Engl..

[14] Wuytens P.C., Subramanian A.Z., De Vos W.H., Skirtach A.G., Baets R. (2015). Gold nanodome-patterned microchips for intracellular surface-enhanced Raman spectroscopy. Analyst.

[15] Wuytens P.C., Demol H., Turk N., Gevaert K., Skirtach A.G., Lamkanfi M., Baets R. (2017). Gold nanodome SERS platform for label-free detection of protease activity. Faraday Discuss..

[16] Otto A., Mrozek I., Grabhorn H., Akemann W. (1992). Surface-enhanced Raman scattering. J. Phys. Condens. Matter.

[17] Moskovits M. (1985). Surface-enhanced spectroscopy. Rev. Mod. Phys..

[18] Itah T., Yamamoto Y.S. (2015). Why and how do the shapes of surface-enhanced Raman scattering spectra change? Recent progress from mechanistic studies. J. Raman Spectrosc..

[19] Yamamoto Y.S., Ozaki Y., Itoh T. (2014). Recent progress and frontiers in the electromagnetic mechanism of surface-enhanced Raman scattering. J. Photochem. Photobiol..

[20] Mosier-Boss P.A. (2017). Review of SERS Substrates for Chemical Sensing. Nanomaterials.

[21] Hulteen J.C., Van Duyne R.P. (1995). Nanosphere lithography: A materials general fabrication process for periodic particle array surfaces. J. Vac. Sci. Technol..

[22] Stuart D.A., Yonzon C.R., Zhang X., Lyandres O., Shah N.C., Glucksberg M.R., Walsh J.T., Van Duyne R.P. (2005). Glucose Sensing Using Near-Infrared Surface-Enhanced Raman Spectroscopy:  Gold Surfaces, 10-Day Stability, and Improved Accuracy. Anal. Chem..

[23] Farcau C., Astilean S. (2010). Mapping the SERS Efficiency and Hot-Spots Localization on Gold Film over Nanospheres Substrates. J. Phys. Chem. C.

[24] Tabatabaei M., Sangar A., Kazemi-Zanjani N., Torchio P., Merlen A., Lagugné-Labarthet F. (2013). Optical Properties of Silver and Gold Tetrahedral Nanopyramid Arrays Prepared by Nanosphere Lithography. J. Phys. Chem. C.

[25] Huebner U., Weber K., Cialla D., Haehle R., Schneidewind H., Zeisberger M., Mattheis R., Meyer H.G., Popp J. (2012). Microfabricated polymer-substrates for SERS. Microelectron. Eng..

[26] Peyskens F., Subramanian A.Z., Neutens P., Dhakal A., Van Dorpe P., Le Thomas N., Baets R. (2015). Bright and dark plasmon resonances of nanoplasmonic antennas evanescently coupled with a silicon nitride waveguide. Opt. Express.

[27] Benabid F., Knight J.C., Antonopoulos G., Russell P.S.J. (2002). Stimulated Raman scattering in hydrogen-filled hollow-core photonic crystal fiber. Science.

[28] Dhakal A., Subramanian A.Z., Wuytens P., Peyskens F., Le Thomas N., Baets R. (2014). Evanescent excitation and collection of spontaneous Raman spectra using silicon nitride nanophotonic waveguides. Opt. Lett..

[29] Boerkamp M., van Leest T., Heldens J., Leinse A., Hoekman M., Heideman R., Caro J. (2014). On-chip optical trapping and Raman spectroscopy using a TripleX dual-waveguide trap. Opt. Express.

[30] Holmstrom S.A., Stievater T.H., Kozak D.A., Pruessner M.W., Tyndall N., Rabinovich W.S., McGill R.A., Khurgin J.B. (2016). Trace-gas raman spectroscopy using functionalized waveguides. Optica.

[31] Evans C.C., Liu C., Suntivich J. (2016). TiO_2_ Nanophotonic sensors for efficient integrated evanescent Raman spectroscopy. ACS Photonics.

[32] Measor P., Seballos L., Yin D., Zhang J.Z. (2007). On-chip surface-enhanced Raman scattering detection using integrated liquid-core waveguides. Appl. Phys. Lett..

[33] Lin S., Zhu W., Jin Y., Crozier K.B. (2013). Surface-Enhanced Raman Scattering with Ag Nanoparticles Optically Trapped by a Photonic Crystal Cavity. Nano Lett..

[34] Kong L., Lee C., Earhart C.M., Cordovez B., Chan J.W. (2015). A nanotweezer system for evanescent wave excited surface enhanced Raman spectroscopy (SERS) of single nanoparticles. Opt. Express.

[35] Peyskens F., Dhakal A., Van Dorpe P., Le Thomas N., Baets R. (2016). Surface Enhanced Raman Spectroscopy Using a Single Mode Nanophotonic-Plasmonic Platform. ACS Photonics.

[36] Peyskens F., Wuytens P., Raza A., Van Dorpe P., Baets R. (2018). Waveguide excitation and collection of surface-enhanced Raman scattering from a single plasmonic antenna. Nanophotonics.

[37] Wuytens P.C., Skirtach A.G., Baets R. (2017). On-chip surface-enhanced Raman spectroscopy using nanosphere-lithography patterned antennas on silicon nitride waveguides. Opt. Express.

[38] Raza A., Clemmen S., Wuytens P., Muneeb M., Van Daele M., Dendooven J., Detavernier C., Skirtach A., Baets R. (2018). ALD assisted nanoplasmonic slot waveguide for on-chip enhanced Raman spectroscopy. APL Photonics.

[39] Tang F., Adam P.M., Boutami S. (2016). Theoretical investigation of SERS nanosensors based on hybrid waveguides made of metallic slots and dielectric strips. Opt. Express.

[40] Li S., Xia L., Chen X., Yang Z., Li W. (2019). Surface-enhanced Raman scattering sensor based on hybrid deep slot waveguide on an integrated photonic platform. J. Opt. Soc. Am. B.

[41] Wong H.M.K., Dezfouli M.K., Sun L., Hughes S., Helmy A.S. (2018). Nanoscale plasmonic slot waveguides for enhanced Raman spectroscopy. Phys. Rev. B.

[42] Cao Q., Feng J., Lu H., Zhang H., Zhang F., Zhang H. (2018). Surface-enhanced Raman scattering using nanoporous gold on suspended silicon nitride waveguides. Opt. Express.

[43] Raza A., Clemmen S., Wuytens P., de Goede M., Tong A.S.K., Le Thomas N., Liu C., Suntivich J., Skirtach A.G., Garcia-Blanco S.M. (2019). High index contrast photonic platforms for on-chip Raman spectroscopy. Opt. Express.

[44] Jun Y.C., Kekatpure R.D., White J.S., Brongersma M.L. (2008). Nonresonant enhancement of spontaneous emission in metal-dielectric-metal plasmon waveguide structures. Phys. Rev. B.

[45] Du P., Zhang X., Yin H., Zhao Y., Liu L., Wu Z., Xu H. (2018). In situ surface-enhanced Raman scattering monitoring of reduction of 4-nitrothiophenol on bifunctional metallic nanostructure. Jpn. J. Appl. Phys..

[46] Mahajan S., Cole R.M., Speed J.D., Pelfrey S.H., Russell A.E., Bartlett P.N., Barnett S.M., Baumberg J.J. (2009). Understanding the Surface-Enhanced Raman Spectroscopy “Background”. J. Phys. Chem. C.

[47] Barnett S.M., Harris N., Baumberg J.J. (2014). Molecules in the mirror: How SERS backgrounds arise from the quantum method of images. Phys. Chem. Chem. Phys..

[48] Hugall J.T., Baumberg J.J. (2015). Demonstrating Photoluminescence from Au is Electronic Inelastic Light Scattering of a Plasmonic Metal: The Origin of SERS Backgrounds. Nano Lett..

[49] Ikeda K., Suzuki S., Uosaki K. (2013). Enhancement of SERS Background through Charge Transfer Resonances on Single Crystal Gold Surfaces of Various Orientations. J. Am. Chem. Soc..

[50] Liu Z., Zhao H., Raza A., Le Thomas N., Baets R. On the Performance of Tantalum Pentoxide and Silicon Nitride Slot Waveguides for On-Chip Raman Spectroscopy. Proceedings of the European Conference on Integrated Optics.

[51] Drag M., Salvesen G.S. (2019). Emerging principles in protease-based drug discovery. Nat. Rev. Drug Discov..

[52] De Gelder J., De Gussem K., Vandenabeele P., Moens L. (2007). Reference database of Raman spectra of biological molecules. J. Raman Spectrosc..

